# Exploring Interactions between Primary Hepatocytes and Non-Parenchymal Cells on Physiological and Pathological Liver Stiffness

**DOI:** 10.3390/biology10050408

**Published:** 2021-05-05

**Authors:** Vaishaali Natarajan, Youra Moeun, Srivatsan Kidambi

**Affiliations:** 1 Department of Chemical and Biomolecular Engineering, University of Nebraska-Lincoln, Lincoln, NE 68588, USA; vaishaali.n@gmail.com (V.N.); youramoeun@gmail.com (Y.M.); 2 The Fred & Pamela Buffett Cancer Center, University of Nebraska Medical Center, Omaha, NE 68198, USA; 3 Nebraska Center for Integrated Biomolecular Communication, University of Nebraska-Lincoln, Lincoln, NE 68588, USA; 4 Nebraska Center for the Prevention of Obesity Diseases, University of Nebraska-Lincoln, Lincoln, NE 68583, USA; 5 Nebraska Center for Materials and Nanoscience, University of Nebraska-Lincoln, Lincoln, NE 68588, USA; 6 Mary and Dick Holland Regenerative Medicine Program, University of Nebraska Medical Center, Omaha, NE 68198, USA

**Keywords:** liver stiffness, hepatocytes, coculture, biomimetic models, cell–cell interaction

## Abstract

**Simple Summary:**

Chronic liver disease is characterized by progressive hepatic fibrosis leading to the formation of cirrhosis irrespective of the etiology with no effective treatment currently available. Liver stiffness (LS) is currently the best clinical predictor of this fibrosis progression irrespective of the cause of the disease. However, it is not well understood how does LS regulate the critical hepatocytes–non parenchymal cell interactions. We here present, to the best of our knowledge, the first analyses of the impact of physiological and pathological stiffness on hepatocytes–non parenchymal cell interaction. Our findings indicate the role of stiffness in regulating the hepatocytes interactions with NPCs necessary for maintenance of hepatocytes function.

**Abstract:**

Chronic liver disease is characterized by progressive hepatic fibrosis leading to the formation of cirrhosis irrespective of the etiology with no effective treatment currently available. Liver stiffness (LS) is currently the best clinical predictor of this fibrosis progression irrespective of the etiology. LS and hepatocytes-nonparenchymal cells (NPC) interactions are two variables known to be important in regulating hepatic function during liver fibrosis, but little is known about the interplay of these cues. Here, we use polydimethyl siloxane (PDMS) based substrates with tunable mechanical properties to study how cell–cell interaction and stiffness regulates hepatocytes function. Specifically, primary rat hepatocytes were cocultured with NIH-3T3 fibroblasts on soft (2 kPa) and stiff substrates that recreates physiologic (2 kPa) and cirrhotic liver stiffness (55 kPa). Urea synthesis by primary hepatocytes depended on the presence of fibroblast and was independent of the substrate stiffness. However, albumin synthesis and Cytochrome P450 enzyme activity increased in hepatocytes on soft substrates and when in coculture with a fibroblast. Western blot analysis of hepatic markers, E-cadherin, confirmed that hepatocytes on soft substrates in coculture promoted better maintenance of the hepatic phenotype. These findings indicate the role of stiffness in regulating the hepatocytes interactions with NPCs necessary for maintenance of hepatocytes function.

## 1. Introduction

Chronic liver diseases affect over 35 million Americans with estimated health care costs of $10 billion per year [1,2,3,4]. Irrespective of the etiology, liver fibrosis is a ubiquitous response with no FDA-approved interventions. Fibroscan measurements have indicated a graded change in liver stiffness (LS) at various stages of fibrosis (2–4 kPa: healthy liver, 8–10 kPa: fibrosis stage of F0–1, 12–25 kPa: F2–4 fibrotic liver, and >55 kPa: cirrhosis) [5,6]. High LS is linked to numerous liver pathologies including cirrhosis, amyloidosis, viral hepatitis, and hepatic carcinoma (HCC) [7,8,9,10,11,12]. Mechanical force across a tissue can change due to fluctuations in blood pressure, the behavior of contractile cells (e.g., hepatic stellate cells-HSCs), and changes in the extracellular matrix (ECM). Following liver injury changes in hepatic blood pressure occur rapidly [13,14], and hypertension in the context of liver disease appears to increase the risk of fibrosis [14,15]. The majority of the emphasis in understanding the role of stiffness during fibrotic liver disease has largely been on HSCs [16,17,18]. However, the impact and the molecular mechanisms that account for the stiffness predilection to hepatocytes dysfunction during fibrosis have been underexplored.

The hepatocytes–non parenchymal cell (NPC) interaction plays a fundamental role in liver function and have been implicated in adult liver physiology and pathophysiology (i.e., cirrhosis and response to injury) [19,20,21]. Liver diseases are perpetuated by the orchestration of hepatocytes and other hepatic non-parenchymal cells (NPCs). Growing evidence shows that under both physiological and pathological conditions, several hepatocyte functions are regulated by neighboring NPCs [22,23,24]. Despite extensive work in addressing the role of hepatocytes interaction with NPCs in regulating hepatic functions, the impact of increasing LS during liver diseases in modulating cell–cell interactions and hepatocyte phenotype in vitro remain unelucidated.

Recent interest in mechanical signaling has led to studying the relationship between stiffness and hepatocyte biology [25,26,27,28,29,30,31]. Dissecting the mechanical microenvironment of physiological and pathological liver stiffness can be challenging in animal models due to their complex nature. As tuning mechanical properties of natural gels is somewhat challenging, several studies have pursued the use of synthetic substrates of varying mechanical properties to examine hepatic phenotype expression [30,32]. Studies have demonstrated that tuning substrate stiffness in combination with the ECM matrix enables regulating hepatocyte function and culture hepatocytes for extended periods [33,34]. In this context, primary hepatocytes grown on increasing film stiffness (elastic modulus of polyelectrolyte multilayers and modified polyacrylamide gels with cell adhesive ligands) are shown to reduce albumin production and impair hepatocytes function [32,35]. Studies have observed that hepatocytes remain growth-arrested and differentiated (functional) on soft environment and proliferate and dedifferentiate (lose their functions) on stiff conditions [36,37,38,39,40]. Hepatocytes cultured on a softer heparin hydrogel (10 kPa) retained five times higher levels of albumin production compared to those on a stiffer heparin gel (110 kPa) after 5 days [34]. We and others have shown that stiffness impedes hepatic urea, albumin production, and expression of drug transporter gene and epithelial cell phenotype marker, hepatocyte nuclear factor 4 alpha (HNF4a) [30,31]. However, a comprehensive understanding of the effect of physiological and pathological stiffness on hepatocytes and NPCs interactions is lacking.

In our study, we utilized a polydimethyl siloxane (PDMS) based substrate with tunable stiffness to study the effect of varying stiffness on hepatocyte-fibroblast heterotypic interactions. We chose the coculture of hepatocytes and NIH 3T3 fibroblasts to model the changes in the heterotypic interactions particularly since they constitute the most utilized culture platform for hepatocytes and coculture with NIH 3T3 has been demonstrated to be a significant inducer of hepatocytes function [41,42,43]. PDMS based substrates are widely used as a biomaterial to study cell–substrate interactions because of its biocompatibility [44,45,46,47], low toxicity [47,48,49], and high oxidative and thermal stability [50,51]. We hypothesize that changes in matrix stiffness will influence hepatocyte–NPC interaction and regulate hepatocyte phenotype and function. To test this hypothesis, we utilized a soft substrate (2 kPa) to represent the healthy liver tissue stiffness and stiff substrate (55 kPa) to represent the diseased liver tissue and compared the cellular properties with the cells grown on collagen coated tissue culture dish (TCPS), which is the gold standard for culturing primary hepatocytes [5,52,53]. Primary rat hepatocytes were then cultured on these gels to reveal that hepatic albumin production, cytochrome activity, and E-cadherin expression were highest on soft gels (2  kPa) when in coculture with fibroblast. Our observations demonstrate a strong dependence of primary hepatocyte function and phenotype on the substrate stiffness thus indicating the crucial role the mechanical environment plays in the regulation of cell–cell communication and in the progression of liver diseases. In the future, identifying stiffness driven mediators of hepatocyte differentiation may have implications for both fundamental hepatology and developing new therapies for liver diseases.

## 2. Materials and Methods

### 2.1. Preparation of PDMS Substrates

**Figure 1:**
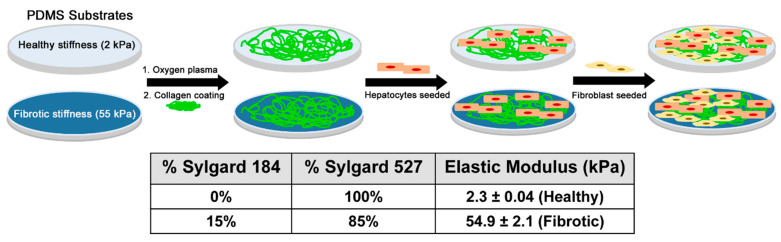
Fabricating gels of varying stiffness by changing the concentration of gel precursor solutions and culturing primary hepatocytes on soft and stiff PDMS gels. Primary rat hepatocytes were seeded on collagen PDMS substrates with physiologically relevant stiffness (2 kPa per soft mimics healthy liver tissue and 55 kPa per stiff mimics diseased/injured liver tissue). After one day in culture, fibroblast is seeded to establish coculture.

Polydimethylsiloxane (PDMS) was used as the substrate to mimic the in vitro healthy and fibrotic liver stiffness. Briefly, Sylgard 527 and Sylgard 184 were taken in the specific weight ratios as mentioned in Figure 1. The crosslinking process was carried following the manufacturer’s guidelines. For Sylgard 527, equal parts of component A and B were mixed well. Sylgard 184 was mixed in a ratio of 10:1 of elastomer to cross-linking agent. The two precursors were then blended in the desired weight ratio (empirically determined) and poured into 12 well tissue culture plates and cross-linking was carried out at 65 °C. Following overnight cross-linking, the 12 well plates were subjected to oxygen plasma treatment for a 7-min duration to render the PDMS surfaces hydrophilic (Plasma Cleaner, PDC001, Harrick Plasma, Ithaca NY, USA) for collagen coating.

### 2.2. Collagen Coating of the Culture Substrates

After overnight crosslinking, the plates containing PDMS substrates were subjected to oxygen plasma treatment for 7 min under the medium RF settings. (Plasma Cleaner PDC-001, Harrick Plasma, Ithaca, NY, USA). The plates were coated with 0.1 mg/mL type 1 collagen solution maintained in 0.02 N acetic acid obtained from rat tail. After overnight incubation at 4 °C, the plates were washed with phosphate buffer saline (PBS) and sterilized under UV.

### 2.3. Isolation and Culture of Primary Hepatocytes

All the animal procedures were carried out in accordance with the guidelines from IACUC of University of Nebraska-Lincoln. Primary rat hepatocytes were isolated from male Sprague-Dawley rats weighing 160–200 g through a two-step collagenase perfusion technique adapted from P.O Seglen [28]. Around 150–200 million cells were obtained at a viability greater than 85% as confirmed by Trypan blue dye exclusion test. Before seeding cells, tissue culture plate surfaces were coated with collagen as described in Section 2.2.

### 2.4. Primary Hepatocyte Culture Medium

Hepatocyte culture medium was freshly prepared with high glucose DMEM supplemented with 10% fetal bovine serum, 0.5 U/mL insulin, 20 ng/mL epidermal growth factor (EGF), 7 ng/mL glucagon, 7.5 mg/mL hydrocortisone, and 1% penicillin-streptomycin. All the constituents for the cell culture medium were obtained from Sigma Aldrich, St. Louis, MO, USA.

### 2.5. Culture of 3T3 Fibroblasts

NIH 3T3 fibroblasts were obtained from ATCC (ATCC CRL-1658) and maintained in culture medium prepared with high glucose DMEM supplemented with 10% fetal bovine serum and 1% penicillin-streptomycin. Approximately 10% of the cells were seeded into a fresh tissue culture flask and the rest of the cells were used for the coculture experiments. Fibroblast medium consisted of DMEM with high glucose, supplemented with 10% bovine calf serum and 200 U/mL penicillin and 200 µg/mL streptomycin.

### 2.6. Coculture on PDMS Surfaces

Six-well plates with PDMS gels of 2 kPa and 55 kPa were coated with collagen and sterilized under UV light overnight. Primary hepatocytes were seeded onto the PDMS surfaces at a cell density of 1.0 × 10^6^/well in a serum-free media for 24 h at 37 °C, 10% CO_2_, balance air. The substrate was then rinsed three times with PBS by pipetting. On the hepatocyte-containing substrates, NIH 3T3 cells were seeded at a density of 0.15 × 10^6^ cells/well and incubated in primary hepatocyte media at 37 °C. The fibroblast/hepatocyte ratio used in this study was 0.15:1, which is the approximate physiologic ratio of stromal and parenchymal cells in the liver [54]. Hepatocyte culture media was used for both monoculture and coculture experiments and fresh media was replenished every 24 h. Phase contrast images of primary hepatocytes cultured on the different substrates were captured using an inverted microscope (Axiovert 40 CFL, Zeiss, Germany).

### 2.7. Differential Trypsinization for Separation of Fibroblast from Primary Hepatocytes

The cells from coculture plates had to be separated before the protein expression of the cell fraction enriched with hepatocytes was analyzed. Primary hepatocytes and fibroblasts were separated from cultures using differential trypsinization in which 1 × trypsin:EDTA was first used to remove the fibroblasts. The primary hepatocytes remained attached to the tissue culture surface and were subsequently detached using 10 × trypsin:EDTA. Cultures were thoroughly observed under a microscope to ensure that only primary hepatocytes remained attached to the substrates following the first trypsinization step. The process of differential trypsinization to separate fibroblasts from other cells have been used widely over several years due to the faster trypsinization of fibroblast [55,56,57,58,59]. To qualitatively validate the selective trypsinization technique, we replated the trypsinized cell suspension onto collagen-coated TCPS and observed that the cell attached were primarily fibroblasts. We used the same established method in our hepatocytes-fibroblast coculture.

The media was carefully collected and spun down to collect the fibroblasts. Hepatocytes on the plate after removal of fibroblasts were lysed with RIPA buffer (for protein collection) and utilized for further analysis.

### 2.8. Urea Assay

Urea secretion by hepatocytes in culture medium was assessed every 24 h using the Stanbio urea nitrogen (BUN) kit (Stanbio, Boerne, TX, USA) using the manufacturer’s protocol. Briefly, the kit exploits the reaction between urea and diacetyl monoxime, which results in a color change at an absorbance of 520 nm read on AD 340 plate spectrophotometer (Beckman Coulter, Brea, CA, USA). Data were expressed as the means ± SD from six independent experiments.

### 2.9. Albumin Assay

Albumin secretion by hepatocytes into culture medium was measured every 24 h using the rat albumin ELISA quantitation kit from Bethyl Laboratories, Inc. (Montgomery, TX, USA) according to the manufacturer’s instructions. In short, a 96 well plate was coated with a coating antibody for 1 h and blocked with BSA for 30 min. The standard/sample was added to each well and incubated for 1 h. HRP detection antibody was incubated for 1 h, followed by the addition of TMB substrate solution that was developed in the dark for 15 min. Absorbance was read on AD340 plate spectrophotometer (Beckman Coulter, Brea, CA, USA) at 450 nm. Data were expressed as the means ± SD from six independent experiments.

### 2.10. Cytochrome P450 Activity Assay

Cytochrome P450 (CYP1A1) activity in primary hepatocytes in all culture conditions was induced using 3-methylcholanthrene (3-MC) at a concentration of 2 µM. The culture media containing 3-MC was replaced every alternate day to induce cytochrome P450 enzyme expression. Before the assay, cells were incubated in 80 µM dicumarol prepared in PBS for 20 min. Ethoxy resorufin o-dealkylase (EROD) activity was measured by incubating cells with phenol red and serum free media containing 5 µM ethoxyresorufin. Cell supernatant was collected at various time points (0, 20, 30, 40, and 50 min). The supernatant was read at an emission of 590 nm and excitation of 530 nm using SLFA plate reader (Biotek, Winooski, VT, USA). Cytochrome activity was calculated as pmol/min and plotted after normalization with respect to the corresponding TCPS monoculture samples.

### 2.11. Western Blot Analysis

Cells were washed with PBS and lysed in 12 well plates containing the PDMS substrates using 75 µL RIPA buffer (100 mM Tris, 5 mM EDTA, 5% NP40) supplemented with 1X protease inhibitor cocktail and phenylmethylsulfonyl fluoride (PMSF) by incubating on ice for ten minutes, followed by the collection of cell lysates in microfuge tubes. Cell debris was pelleted out and supernatants with proteins were stored away at −80 °C until use. Protein concentration was determined through colorimetry using the Pierce™ BCA protein assay kit (Fisher Scientific, Rockford, IL, USA). Protein was loaded onto 10% SDS-containing polyacrylamide gels and after PAGE, were transferred onto Immobilon CL membrane (Millipore, Burlington, MA, USA). Membranes were blocked using 5% skimmed milk for 2 h at room temperature (RT) following which the blots were incubated overnight at 4 °C in anti-E-cadherin antibody (Abcam, CA) or anti-GAPDH (Abcam MA) antibodies. Following the primary antibody incubation, the blots were incubated for one hour at RT in near infrared 680 nm and 800 nm secondary antibody (Fisher Scientific, PA) and signal for protein expression was detected using the Odyssey infrared imaging system (Li-COR Biosciences, Lincoln, NE, USA). Densitometric analysis of the blots was performed using the Image Studio software associated with the Odyssey imaging system.

### 2.12. Statistical Analysis

Data were expressed as the mean ± SD from six independent experiments. The difference between the various experimental groups was analyzed by a one-way analysis of variance (ANOVA) using the statistical analysis embedded in GraphPad Prism Software (San Diego, CA, USA) using a Tukey test. Q tests were employed to identify outliers in the data subsets. For statistical analysis of all data, *p* < 0.05 was used as the threshold for significance.

## 3. Results

This article explores how stiffness affects the phenotype of cultured hepatocytes in coculture with non-parenchymal cells. Primary hepatocytes cultured on softer PDMS gels with a modulus of 2  kPa were more functional than cells on stiffer substrates (55 kPa) as observed by albumin synthesis and E-cadherin expression. This work supports the notion that stiffness represents an important inducer of phenotypes in primary hepatocytes and modulates cell–cell communication critical for hepatocytes function.

### 3.1. Measuring Elastic Modulus of the PDMS Substrates

In this study, the elastic modulus (*E*) of PDMS gels was tuned by controlling the concentration of the crosslinker solution. The modulus was determined using indentation load technique with a nanoindenter. Figure 1 illustrates the impact of the crosslinkers and concentrations of Sylgard on the resulting elastic moduli of PDMS gels. A 100% (*w/w*) Sylgard 527 gel was the softest with an elastic modulus of 2.3 ± 0.04 kPa, whereas the 85% (*w/w*) Sylgard 527 gel and 15% by weight Sylgard 184 was the stiffest with an elastic modulus of 54.9 ± 2.1  kPa. These elastic moduli fall into the physiological liver stiffness range of healthy and diseased liver making them an ideal substrate to investigate the role of liver stiffness on hepatic function [53]. The control used in this study was collagen coated tissue culture dish had elastic modulus of 3 × 10^6^ kPa [60]. The stiffness measured using mechanical indentation methods have been shown to have a linear correlation with stiffness measured by elastography measurements [61,62]. This comparison has also been performed on scaffolds as well to demonstrate that the elastography and indentation-based stiffness measurements correlates well indicating that the stiffness we used are similar to the stiffness of healthy and fibrotic liver from fibroscan measurements.

### 3.2. Primary Hepatocytes/Fibroblast Coculture on PDMS Substrates

**Figure 2:**
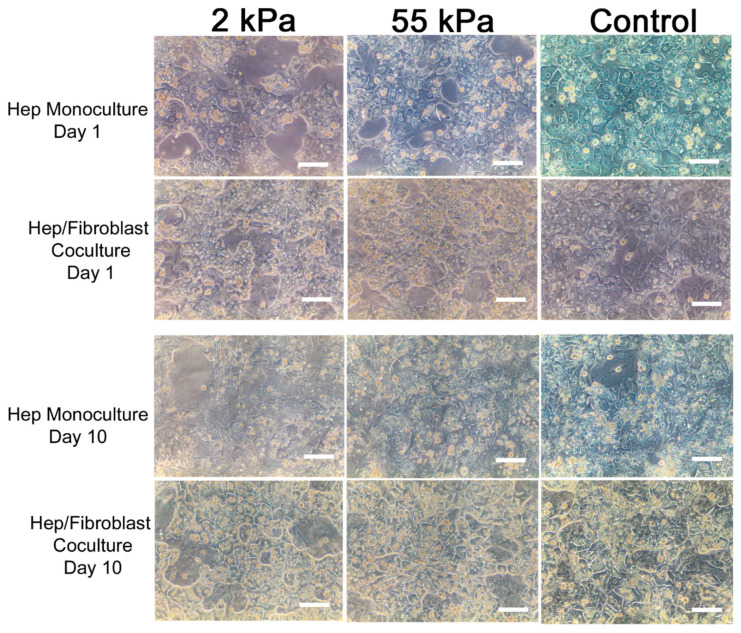
Morphology of primary rat hepatocytes on gels of varying stiffness in the monoculture and coculture. Phase images of hepatocytes cultured on soft (2 kPa), stiff (55 kPa) and TCPS at day 1 and 10. Scale bar = 100 µm.

We investigated the effect of substrate stiffness on the morphology and viability of primary hepatocytes cultured on PDMS substrates coated with collagen and compared the results with petri dish coated with collagen as a control. Figure 2 shows representative images of hepatocytes cultured on PDMS gels of different mechanical properties and coated TCPS (control) at days 1 and 10 as a monoculture and coculture with fibroblast. As seen from these images, hepatocytes in coculture with fibroblast showed better morphology in all three substrates at day 10 and displayed clear cell–cell tight junctions forming the epithelial sheet and polygonal shape of the individual cells. In comparison, hepatocytes monoculture in all three substrates spread out and became less cuboidal—a sign of dedifferentiation and loss of epithelial phenotypes [63,64]. In our previous study, we demonstrated that hepatocytes monoculture on TCPS displayed considerable increase in cell-spreading and a fibroblast-like morphology while 2 kPa and 55 kPa retained the initial polygonal shape and visible tight junctions between the cells [30]. This result suggests that coculture plays a key role in preserving the impact of hepatocytes communication with non-parenchymal cells.

### 3.3. Effect of Stiffness on Primary Hepatocytes Urea Production in the Coculture

**Figure 3:**
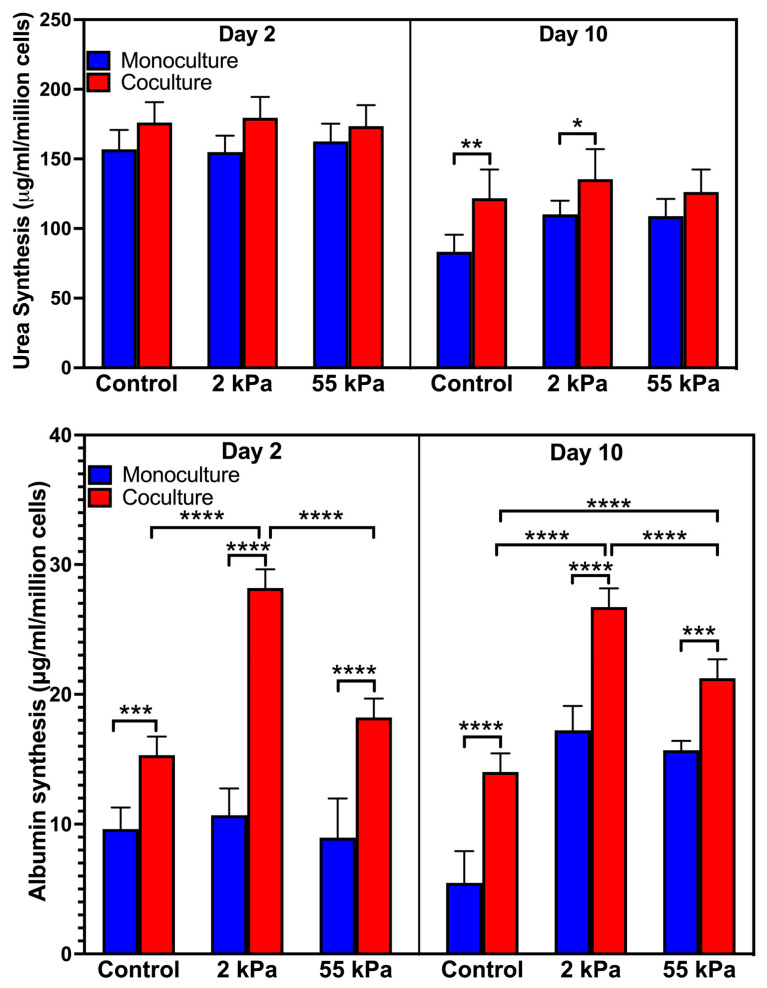
Hepatic urea and albumin expression as a function of gel stiffness in the monoculture and coculture. (**A**) Quantification of urea synthesis by primary hepatocytes on soft (2 kPa), stiff (55 kPa), and TCPS substrates; (**B**) quantification of albumin synthesis primary hepatocytes cultured on soft (2 kPa), stiff (55 kPa), and TCPS substrates. Error bars indicate standard deviation of the mean for *n* = 5 samples. * *p* < 0.05, ** *p* < 0.01, *** *p* < 0.001, **** *p* < 0.0001.

We examined the effect of stiffness in urea synthesis, a key functional marker for primary hepatocytes that is indicative of intact nitrogen metabolism and detoxification (Figure 3A) on days 2 and 10. Hepatocytes in coculture on 2 kPa substrates produced 135.5 ± 21.5 µg/mL/million cells urea on day 10 compared to 126.2 ± 16.3 µg/mL/million cells urea and 121.8 ± 20.6 µg/mL/million cells urea by hepatocytes in coculture on 55 kPa and TCPS substrates on day 10, respectively. The urea production in 2 kPa (110.2 ± 9.8 µg/mL/million cells) and TCPS (83.3 ± 12.2 µg/mL/million cells) in the monoculture were significantly lower than hepatocytes cultured in the coculture while there was no significant difference in urea production in hepatocytes in the monoculture and coculture on 55 kPa.

### 3.4. Effect of Stiffness on Primary Hepatocytes Albumin Synthesis in Coculture

We next examined the effect of stiffness in albumin synthesis, which is a widely accepted marker of hepatocyte synthetic function (Figure 3B) on days 2 and 10 [37,38]. On day 2 in coculture, hepatocytes on 2 kPa coculture produced 28.2 ± 1.43 µg/mL/million cells whereas the hepatocytes in coculture on 55 kPa produced 18.2 ± 1.45 µg/mL/million cells and TCPS had 15.3 ± 1.43 µg/mL/million cells albumin, respectively. Hepatocyte monocultures in all three substrates had similar albumin production and were the least. On day 10, the hepatocytes in the coculture on 2 kPa had the highest albumin production (26.7 ± 1.44 µg/mL/million cells) and comparable to its day 2 values while the hepatocytes in the coculture in 55 kPa (21.2 ± 1.74 µg/mL/million cells) and control (14.0 ± 1.94 µg/mL/million cells) had lower albumin production. This result shows that stiffness plays a key role in maintaining hepatocytes albumin function in the coculture systems as well.

### 3.5. Effect of Stiffness on Hepatocytes CYP1A1 Activity in Coculture

**Figure 4:**
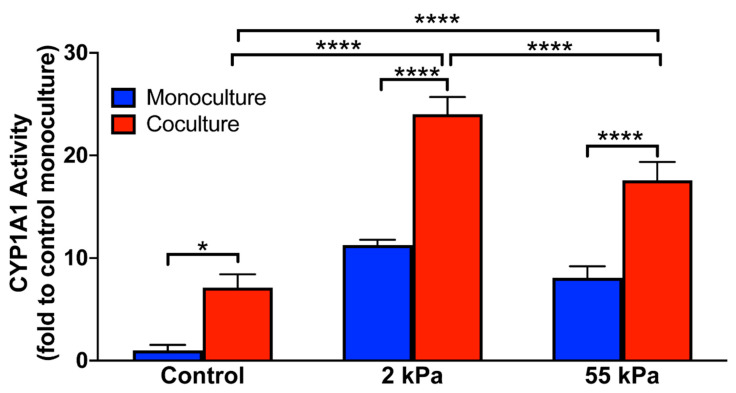
Quantification of cytochrome P450 activity of primary hepatocytes when cultured on soft, stiff and TCPS substrates on day 10 of culture. Error bars indicate standard deviation of the mean for *n* = 5 samples. * *p* < 0.05, **** *p* < 0.0001.

We induced cytochrome P450 enzyme expression in primary hepatocytes by treating them with 3-methylcholanthrene and evaluated the enzyme activity using the substrate ethoxyresorufin. As seen in Figure 4, we observed that hepatocytes in coculture on the 2 kPa matrix after 10 days in culture had over 25 folds higher enzyme activity than hepatocytes cultured in the monoculture on the control. We also observed that among coculture samples, the 2 kPa matrix supported the functional maintenance of hepatocytes best, followed by the 55 kPa substrate. Although coculture on the control displayed roughly 9 folds higher cytochrome activity when compared with their monoculture counterparts, the control coculture retained less than 50% of the function of the 2 kPa coculture. CYP1A1 activity on hepatocytes in monoculture on 2 kPa and 55 kPa on day 10 was 11.3 and 8.1 fold higher than TCPS, respectively. Furthermore, the CYP activity of hepatocytes on 2 kPa on day 10 was significantly higher than the cells on 55 kPa (statistics data not shown in graph). This is akin to our previous study where we demonstrated that stiffness alone regulates CYP1A1 activity [30]. These results in the current study suggest that hepatocytes interaction with non-parenchymal cells and stiffness both collectively regulate the hepatic metabolic functions.

### 3.6. Effect of Stiffness Primary Hepatocytes E-Cadherin Expression in Coculture

**Figure 5:**
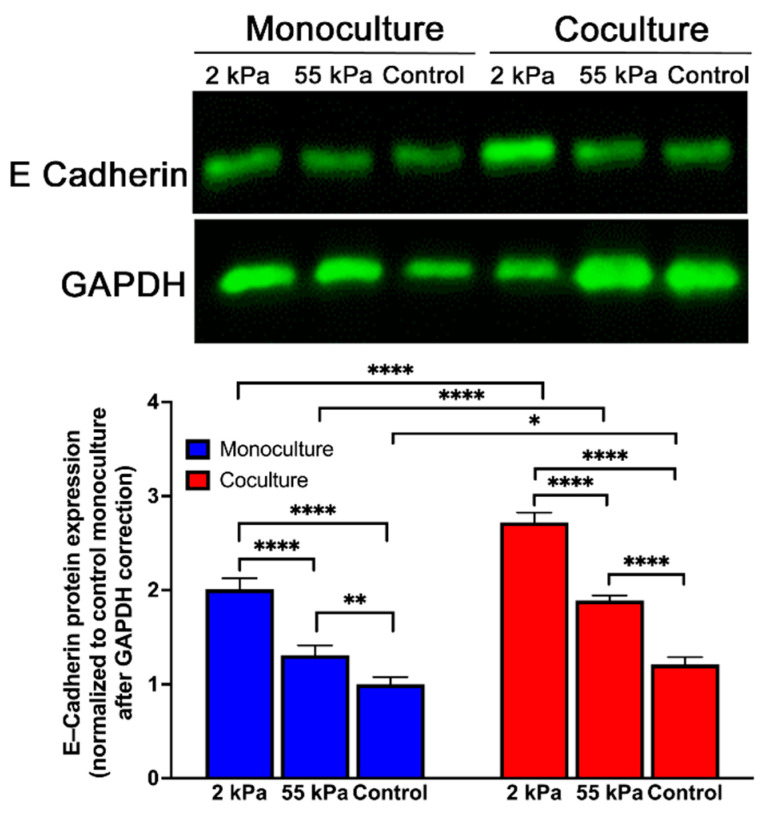
Expression of E-cadherin in hepatocytes cultured on PDMS gels. Western blot and quantification of e-cadherin expression of primary hepatocytes when cultured on soft, stiff, and TCPS substrates. Error bars indicate standard deviation of the mean for *n* = 3 samples. * *p* < 0.05, ** *p* < 0.01, **** *p* < 0.0001. The representative blot does not accurately represent the quantitative mean data shown.

To further analyze hepatic function on different stiffness, we investigated the expression of E-cadherin—an epithelial marker expressed by differentiated hepatocytes. As shown in Figure 5 and Figure S1, E-cadherin expression was significantly higher on 2 kPa substrates in coculture compared to 55 kPa and control substrates. In addition, the cocultures overall had higher E-cadherin expression in all substrates compared to their corresponding monocultures. Overall, analysis of albumin synthesis and E-cadherin expression suggest that the hepatic phenotype was maintained better on the softer matrix that recreates physiological liver stiffness. This result confirms that stiffness is key in retaining the positive effects of coculture on hepatocytes function and maintenance in culture.

## 4. Discussion

Heterotypic cell–cell interactions between hepatocytes and NPCs are vital in the maintenance of hepatocyte functions. The complex interplay between the parenchyma and non-parenchymal cells changes drastically in the event of liver diseases. There is a critical need to engineer in vitro models that will mimic the various stages of liver disease to serve as accurate models for studying disease mechanism and drug and toxicity testing. Such models need to incorporate the dynamic changes in the liver microenvironment including the change in LS. In this study we aimed to (1) determine the combined role of mechanical stiffness and coculture mediated cell–cell contact in regulating functional stability of hepatocytes and (2) create an in vitro model of the fibrotic liver to study the nature of paracrine interaction between various liver cell types. Primary hepatocytes are notoriously challenging to culture in vitro and rapidly dedifferentiate resulting in a complete loss in phenotype in about 5 days in culture [25]. We applied this model to the well characterized coculture of hepatocytes and fibroblasts and our preliminary results suggest that by combining the two major liver microenvironment aspects of the healthy liver namely heterotypic cell interaction and matrix stiffness, hepatocyte function can be maintained efficiently for at least 10 days.

Biomaterial substrate used for the in vitro model through the physicochemical properties can impact cell behavior ranging from attachment, proliferation, and function [26,27]. In the model described here, hepatocyte attachment to the substrate was maintained for longer time periods in the coculture setting compared to the monoculture across all conditions (2 kPa and 55 kPa) and provided an insight toward the cells behavior when grown on healthy and disease liver microenvironment. Researchers have relied on hepatocyte mediated urea and albumin synthesis for evaluating the synthesis and metabolic functions of these cells in vitro [28]. Our results indicate that urea and albumin synthesis both are influenced by matrix stiffness and presence of fibroblasts in the culture. Albumin synthesis, particularly, was found to be highly inducible by the presence of these two favorable cues. Thus, we believe that stiffness and cell–cell interaction may indeed play a key role in modulating hepatocytes phenotype and function.

In vitro liver models are highly valuable towards modeling the xenobiotic metabolism function of hepatocytes to study the drug mediated hepatotoxicity to the liver [29]. Our model demonstrates that by recreating the mechanical environment of healthy liver and coculturing hepatocytes and fibroblasts on this substrate, the cytochrome activity can be up to 25 folds higher even after 10 days in culture. This is an important finding since based on the drug catabolizing machinery in vitro, the dose response of novel drugs can appear entirely different and alter the reliability of in vitro tests. Similar finding was observed in the case of hepatocarcinoma cells where matrix compliance was found to alter the chemotherapeutic drug tolerance levels [30,31].

Another important aspect of this study is the optimization of the differential trypsinization procedure for separating hepatocytes and fibroblasts from the coculture system. The most popular technique for cell sorting is fluorescence activated cell sorting (FACS) that requires the use of at least one type of monoclonal antibody for a cell-specific antigen [32]. Primary prerequisite for FACS is the presence of reliable cell surface markers and fibroblasts are notoriously devoid of specific markers. Although the purity is generally higher, FACS also presents disadvantages such as high costs, requirement of high cell number, and availability of instruments. Fibroblast attachment to substrates is comparatively less robust as opposed to hepatocytes and we exploited this aspect to separate the two cell populations based on differential trypsinization time. The process of differential trypsinization to separate fibroblasts from other cells have been used widely over several years due to the faster trypsinization of fibroblast [55,56,57,58,59].

Morphology of hepatocytes is the primary indicator of the highly differentiated phenotype of hepatocytes and our study shows that the in vitro model of hepatocytes and fibroblasts cocultured on 2 kPa matrix, demonstrate superior morphological integrity. Maintenance of tight cell–cell junctions between hepatocytes also reflected in E-cadherin protein expression. In case of normal epithelium in vivo, adherens-type junctions stabilize the interactions between adjacent cells [33]. The cytoplasmic domain of these adherens structure is formed by cadherins, which interact with cytoskeletal elements to regulate a cascade of cellular events. Higher expression of E-cadherin in our model system is a potential regulator of the functional maintenance that is observed. Additionally, loss in E-cadherin expression is considered synonymous with development of disease-like phenotype in hepatocytes [34,35].

Existing liver platforms overlook the hepatocyte-non parenchymal cell communication in combination with mechanical environment. While the older two-chamber cocultures of donor and recipient cells separated by a pore membrane is widely used as a physiological system for the investigation of intercellular communication, it physically separates the different cell types used without direct cell–cell interaction. Further, in the indirect coculture, signaling between different cell types occurs chiefly through paracrine effects via soluble factors. Notably, the cascade processes such as migration, proliferation, and differentiation require direct intercellular contact-based cell–cell communication and signaling reactions that operate in a paracrine/autocrine loop or fashion. The closest we have to a liver culture system that exhibits these processes is 3D organoids cultured from primary hepatocytes/induced pluripotent stem cells derived liver cells [65,66,67]. However, they are limited as they do not contain other cells types in the liver to study the hepatocyte–non parenchymal interaction; dependent on animal-based matrix that has batch to batch variability; challenging to modulate mechanical environment without changing the physical properties such as porosity and diffusion, which will regulate cell behavior.

Our in vitro model can be applied towards coculture of other hepatic cell types that play an important role in liver physiology such as hepatocytes-stellate cells, hepatocytes-LSECs. The in vitro platform allows for a systematic analysis of the molecular mechanisms that influence the cell types in coculture and the mechanical component of the system allows for mimicry of the environment of healthy and fibrotic liver. Future studies utilizing sequencing based approaches can provide further insight into the identity of various paracrine factors and microvesicles that fibroblasts release on the 2 kPa (healthy) matrix that enables improved functional support of hepatocytes. Additionally, identifying stiffness driven mediators of hepatocyte differentiation may have implications for both fundamental hepatology and developing new therapies for liver diseases.

## 5. Conclusions

The present study shows that substrate modulus and cell–cell interactions both regulate hepatocyte function. The production of urea and albumin was affected by both substrate stiffness and cell–cell interaction, with high expression requiring both cell contact and softer substrate. Our experiments also documented that hepatocytes expressed higher levels of E-cadherin, on the softer substrate (2 kPa) when in coculture with a fibroblast. Our findings pointing to the importance of substrate mechanical properties on hepatocyte function point to the critical role of LS just not a consequence but also cause of liver fibrosis and hepatocyte dysfunction.

## Data Availability

Data is contained within the article

## Supplementary Materials

The following are available online at https://www.mdpi.com/article/10.3390/biology10050408/s1, Figure S1: Full blots of Figure 5.
