# Supplementary figures and images for: Exploring Interactions between Primary Hepatocytes and Non-Parenchymal Cells on Physiological and Pathological Liver Stiffness
Source: Biology (Basel). 2021 May 5;10(5):408. doi: 10.3390/biology10050408 (PMC8147966; doi:10.3390/biology10050408)

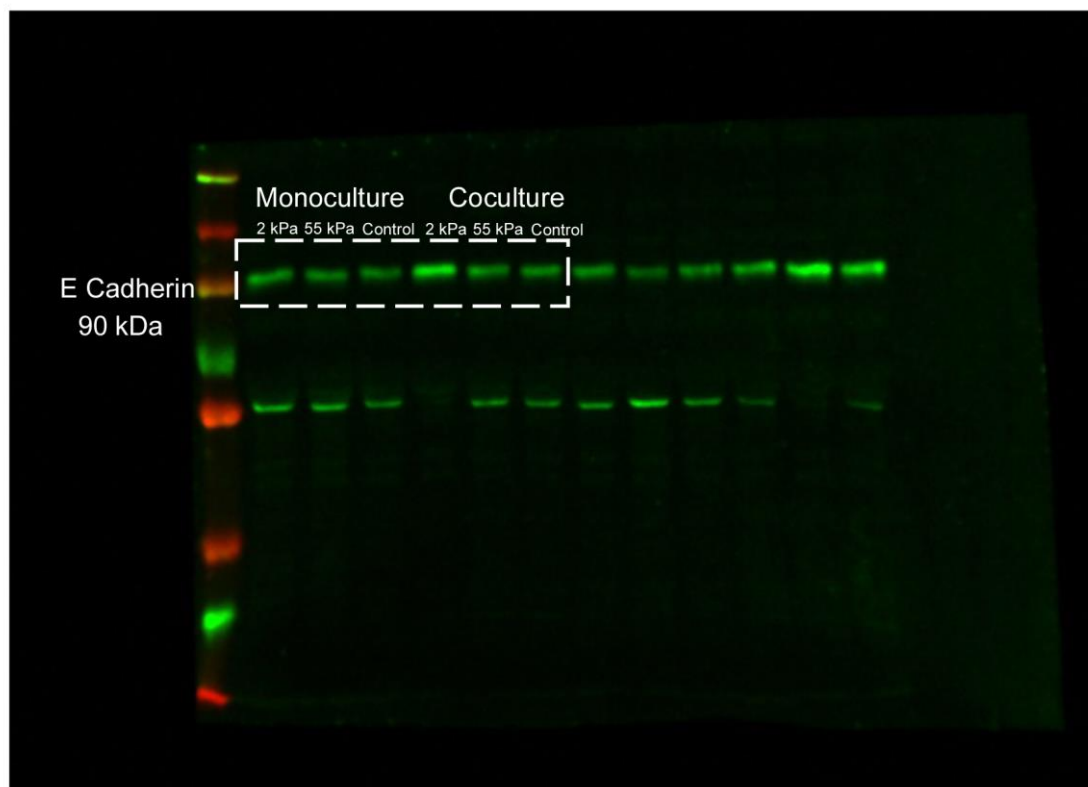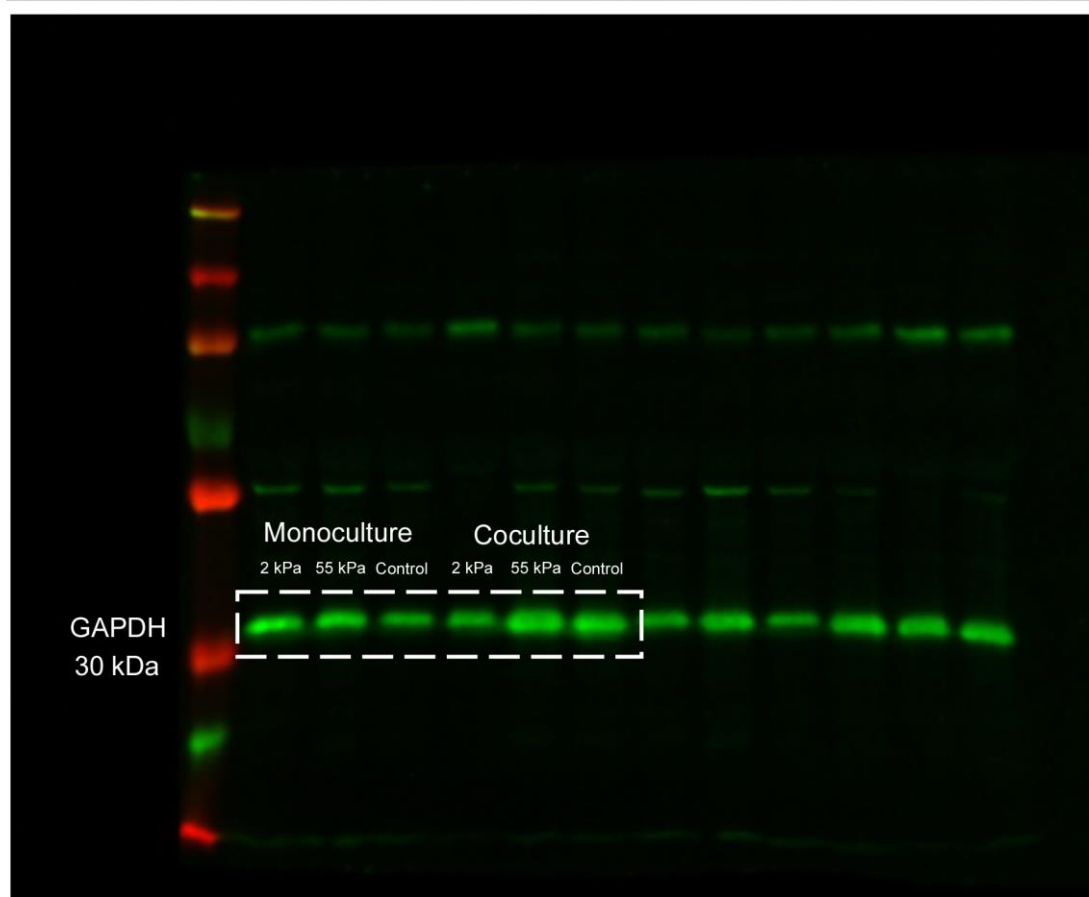

Figure S1. Full blots of Figure 5.

Supplement: Supplementary file 1 [file biology-10-00408-s001.zip › biology-1140043-supplementary.pdf]
